# The Role of nAChR and Calcium Signaling in Pancreatic Cancer Initiation and Progression

**DOI:** 10.3390/cancers7030845

**Published:** 2015-07-31

**Authors:** Courtney Schaal, Jaya Padmanabhan, Srikumar Chellappan

**Affiliations:** 1 Department of Tumor Biology, H. Lee Moffitt Cancer Center and Research Institute, 12902 Magnolia Drive, Tampa, FL 33612, USA; E-Mail: courtney.schaal@moffitt.org; 2 Department of Molecular Medicine and USF Health Byrd Alzheimer’s Institute, University of South Florida, 4001 E. Fletcher Ave., Tampa, FL 33612, USA; E-Mail: Jpadmana@health.usf.edu

**Keywords:** nicotine, tobacco carcinogens, calcium channels, beta-adrenergic receptors, epithelial mesenchymal transition, metastasis

## Abstract

Pancreatic cancer shows a strong correlation with smoking and the current therapeutic strategies have been relatively ineffective in improving the survival of patients. Efforts have been made over the past many years to understand the molecular events that drive the initiation and progression of pancreatic cancer, especially in the context of smoking. It has become clear that components of tobacco smoke not only initiate these cancers, especially pancreatic ductal adenocarcinomas (PDACs) through their mutagenic properties, but can also promote the growth and metastasis of these tumors by stimulating cell proliferation, angiogenesis, invasion and epithelial-mesenchymal transition. Studies in cell culture systems, animal models and human samples have shown that nicotinic acetylcholine receptor (nAChR) activation enhances these tumor-promoting events by channeling signaling through multiple pathways. In this context, signaling through calcium channels appear to facilitate pancreatic cancer growth by itself or downstream of nAChRs. This review article highlights the role of nAChR downstream signaling events and calcium signaling in the growth, metastasis as well as drug resistance of pancreatic cancer.

## 1. Introduction

Pancreatic cancer is the fourth leading cause of cancer deaths in both men and women in the US [1], with an estimated 48,960 new cases and 40,560 deaths predicted for 2015 alone. Due to the lack of effective early detection methods, most pancreatic cancers present at late stages when the disease is locally advanced or has already metastasized, resulting in poor prognosis and a low 5-year survival rate of 7.5% [1]. Pancreatic ductal adenocarcinoma (PDAC) comprises 90% of all pancreatic cancer cases, and is an exocrine tumor arising from ductal or acinar cells [2] with a mortality rate of nearly 100% within 2 years, a statistic which has not declined over the past decades despite growing knowledge of the disease [3]. Gemcitabine alone or in combination with other agents has been the standard of care for advanced PDAC; however, little progress has been made in effective treatment options due to the highly resistant nature of pancreatic cancer.

Epidemiological studies have provided strong evidence that tobacco smoke increases the risk of pancreatic cancer in a dose and exposure-dependent manner [4,5,6,7]; in a meta-analysis of tobacco risk and pancreatic cancer from 82 studies, there was an estimated 75% increased risk for pancreatic cancer in smokers *versus* non-smokers. While smoking cessation can lower this risk by up to 50%, the overall risk compared to non-smokers remains elevated in former smokers for up to 10 years or more [8,9]. In general, it is believed that smoking accounts for 20%–30% of all pancreatic cancer cases, and is the most preventable cause of the disease. Tobacco smoke contains at least 60 known carcinogens, which have been shown to initiate tumorigenesis [10,11]. This occurs primarily through the formation of DNA adducts resulting in mutations in a number of vital genes implicated in tumorigenesis, including KRAS, which is mutated in nearly all pancreatic cancers [12,13,14].

Nicotine, the addictive component of tobacco smoke, is generally thought to be non-carcinogenic, though recent studies have suggested that chronic exposure at high concentrations can initiate muscle sarcomas in A/J mice [15,16]. At the same time, nicotine has been shown to enhance cell proliferation, migration, invasion, and angiogenesis in multiple cancer types, including those of the pancreas [17,18,19,20], thus acting as a tumor promoter [18]. Additionally, nicotine has been shown to disrupt metabolic processes and confer drug resistance to cancer cells. The primary mechanism by which nicotine exerts its tumor promoting effects is through the binding to and activation of nicotinic acetylcholine receptors (nAChRs) [21,22,23] and to some extent β-Adrenergic receptors (β-ARs) [21,24]. Upon binding to these cell surface receptors, a number of signaling cascades are initiated which result in tumor progression [21,23]. The nicotine derivatives *N*-nitrosonornicotine (NNN) and 4-(methylnitrosamino)-1-(3-pyridyl)-1-butanone (NNK) have also been found to bind to nAChRs with high affinity stimulating multiple tumor promoting signaling cascades, and unlike nicotine its two derivatives can exert carcinogenic effects through the binding of these receptors as well as by inducing DNA damage [21,25,26]. nAChRs are pentameric cell surface receptors typically expressed in the central and peripheral nervous systems and at neuromuscular junctions where they act to facilitate signal transduction as well as calcium influx resulting in the release of neurotransmitters such as dopamine, serotonin, γ-aminobutyric acid (GABA), and the catecholamine neurotransmitters noradrenaline and adrenaline [27,28]. The synthesis and release of catecholamines results in the binding to and activation of β-adrenergic receptors, which in turn activate a number of additional signaling pathways including those resulting in cyclic AMP (cAMP) formation [29]. More recently, both nAChRs and β-ARs have been found to be expressed on cells of epithelial and endothelial origin including pancreatic cells and pancreatic cancer cells, where they act to initiate intracellular signaling cascades and mediate the synthesis and release of multiple growth, angiogenic, and neurotrophic factors [21,30,31,32].

Studies in NSCLCs have shown that nicotine promotes the invasive nature of cancer cells and these effects of nicotine are mediated through α7-nAChR [17,23]. The tobacco-specific nitrosamines and nicotine are known to increase calcium signaling in cells [33,34,35]; exposure to nicotine has been shown to consistently evoke transient Ca responses, which are selectively inhibited by α7-nAChR antagonists, methyllycaconitine or α-bungarotoxin, and are greatly increased by the allosteric modulator PNU-120596 [36,37,38]. Different nAChR subunits allow differential permeability in calcium, with α7-nAChR showing the highest permeability [39]. In addition to its direct influence on calcium influx, nAChRs are also known to induce calcium levels through activation of voltage-dependent calcium channels (VDCCs) and calcium-induced calcium release (CICR) from the endoplasmic reticulum though ryanodine receptors (RyRs) and inositol (1,4,5)-triphosphate receptors (IP3Rs) [40]. Since calcium plays an important role in the normal functioning of the cells, it can be imagined that alteration in intracellular calcium levels will affect normal cellular functions and promote proliferation, motility, invasion and survival, thus affecting the efficacy of anti-cancer therapies. Elucidating the role of calcium signaling and the mechanisms by which nicotine and nAChRs enhance tumor progression might lead to the development of more effective treatment modalities for smoking related pancreatic cancers, for which there are currently no effective treatment options.

## 2. Nicotine and Pancreatic Cancer Development

Pancreatic cancers encompass both malignancies of the exocrine and neuroendocrine components, and while endocrine tumors account for approximately 5% of pancreatic cancers, exocrine pancreatic ductal adenocarcinomas or PDACs account for 90% of the cases. PDACs originate from ductal cell lineage or acinar cells that undergo acinar-to-ductal metaplasia, and most often they arise from pancreatic intraepithelial neoplasms (PanINs) associated with pancreatic inflammation [2]. Loss of pancreatic acinar cell differentiation has also been linked to increased metaplasia [41].

In a mouse model where tumorigenesis was initiated by implanting 7,12-dimethylbenzanthracene (DMBA) crystals into the pancreas, it was found that nicotine could promote PanIN lesion development, as well as development of adenocarcinomas [42]. Activating KRAS mutations are thought to be a critical initiating step in the development of PDAC [43] and such mutations can be induced by tobacco carcinogens: additionally, the rate of KRAS mutations in the pancreas increases with age [44]. In KRAS G12V as well as KRAS/P53 mouse models of pancreatic cancer, it was found that mice treated with nicotine for 86 weeks had increased incidence of low grade PanIN lesions and increased acinar-to-ductal metaplasia with atrophy compared to control mice; furthermore, only mice treated with nicotine developed high grade PanIN lesions [45]. These results were consistent with those reported in a KRAS G12D mouse model of pancreatic cancer, where mice exposed to total cigarette smoke for 20 days had increased PanIN formation, and concomitantly showed an increase in the ductal marker CK19 and a decrease in the acinar marker amylase, suggesting enhanced acinar-to-ductal metaplasia [46].

Nicotine treatment of KRAS G12V and KRAS/P53 mice for 86 days led to a marked reduction in expression of GATA6 and Mist1 genes, which are known regulators of acinar cell differentiation; and this coincided with decreased acinar cell granularity and enzyme production [45]. This effect occurred through the nicotine-mediated activation of α7 nAChR, and subsequent activation of AKT and ERK1/2 signaling pathways resulting in activation of C-Myc and repression of GATA6 gene expression [45]. In addition to nicotine inducing acinar cell de-differentiation in these models, it was shown that nicotine could enhance pancreatic progenitor cell activity through upregulation of the stem cell genes/markers Sox9 and ALDH, and could also enhance pancreatic cancer stem cell populations through the upregulation of Sox9, Oct3/4, and ALDH [45]. Further, nicotine could enhance self-renewal *in vitro* as well as tumorigenecity and cancer stem cell frequency *in vivo* [45]. The stem cell promoting effects of nicotine were also found to occur through the nicotine-mediated activation of α7 nAChR, and subsequent activation of AKT and ERK1/2 [45]. Since pancreatic cancer stem cells are thought to be responsible for the initiation and maintenance of PDACs, the above studies suggest that nicotine enhances the tumor initiating functions of the stem cells, promoting the development of pancreatic cancer.

### 2.1. Nicotine Mediated Alterations in Gene Expression Profiles

Exposure to nicotine has been shown to alter gene expression profiles of various cancers in different systems [47,48]. Attempts have been made recently to evaluate cigarette smoking and its association with genome-wide alterations in pancreatic cancer [49,50]. Analysis of 2028 cases and 2109 controls from a genome-wide association study (GWAS) and risk factor data from the Pancreatic Cancer Case Control Consortium was conducted at the pathway/gene/single nucleotide polymorphism level [50]. The top two pathways identified in this analysis included the pancreatic secretion and salivary secretion pathways; Ingenuity Pathway Analysis (IPA) identified genes involved with axonal guidance and α-adrenergic signaling to have interactions with smoking [50]. SLIT/ROBO signaling genes, which are involved in the axon guidance pathways, were further identified to be frequently altered in pancreatic cancer [50]. Interestingly, there was no noted association with nicotine dependence genes or pathways, or with genes found to be associated with smoking in other cancer types [50]. The reason for this discrepancy with studies on nicotine and tobacco carcinogen-mediated gene expression studies in mouse and cell culture experimental systems and human patient samples is not known.

In addition to the association of smoking and pancreatic cancer at the genome level, it has been reported that nicotine has an effect on pancreatic cells at the proteome level as well. The impact of nicotine on the proteome was studied in both a normal immortalized pancreatic ductal cell line HPNE, as well as in a malignant pancreatic cancer cell line Panc1 using mass spectrometry. In this study, over 900 proteins were found to be differentially upregulated or downregulated with nicotine exposure [51]. Of these, 57 proteins were found to be altered in both the normal and cancer cell lines but the type of alterations varied between cell types [51]. Cell surface receptor signal transduction proteins were upregulated in both cell types; membrane proteins involved in cell adhesion, transmembrane ion transport, and oxidative phosphorylation were down regulated in Panc1 cells. At the same time, proteins involved in cytokine response, steroid biosynthesis, cell migration, extracellular structure organization, and JAK/STAT pathway were found to be upregulated in HPNE cells while proteins involved in cell division, ribosomes, and cell cycle were downregulated in HPNE but upregulated in Panc1. Interestingly, nAChR binding proteins including amyloid precursor protein (APP) were upregulated in both cell types [51]. The APP protein, which is associated with Alzheimer’s disease, is expressed at high levels in the pancreas, can bind to α7 nAChR and has been previously shown to be increased in nicotine treated pancreatic cells across multiple species (mice, rat, and human), suggesting that it could potentially play a role in nicotine-mediated progression of pancreatic cancer, but this warrants further studies [51].

### 2.2. nAChR Mediated Proliferative and Invasive Signaling Events

**Figure 1:**
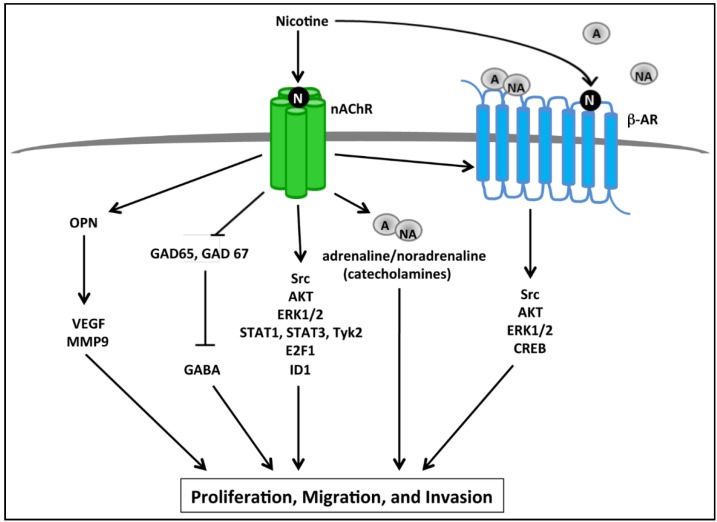
Schematic representing downstream pathways activated by nicotine that promote proliferation, migration, and invasion of pancreatic cancer cells. Activation of nAChRs upon nicotine binding results in the activation of multiple signaling cascades as well as the release of catecholamines such as adrenaline (A) and noradrenaline (NA). Adrenaline and noradrenaline in turn bind to and activate β-adrenergic receptors (β-ARs), resulting in activation of Src kinase, and subsequent activation of AKT, ERK1/2, and CREB leading to enhanced proliferation of pancreatic cancer cells. nAChR stimulation results in increased osteopontin (OPN), which then increases VEGF and MMP9 levels resulting in increased proliferation and migration of pancreatic cancer cells. Similarly, nicotine represses GAD65 and GAD67 enzymes which are responsible for the synthesis of GABA, which typically acts to repress pancreatic cancer cell proliferation, migration, and invasion; therefore nicotine can promote proliferation by suppressing GABA synthesis. Further, nicotine is known to bind to nAChRs and activate Src, AKT, and ERK1/2 signaling proteins, as well as lead to the activation of STAT1, STAT3, E2F1, and ID1 transcription factors, resulting ultimately in increased pancreatic cancer cell proliferation, migration, and invasion.

Analogous to the function of nAChRs in the nervous system, it was found that nAChRs could regulate the synthesis and release of catecholamines in both normal pancreatic ductal cells as well as cancer cells, resulting in an autocrine catecholamine loop responsible for the activation of a number of signaling pathways that are elevated in pancreatic cancer [52,53,54]. It was further demonstrated that acute nicotine treatment induced secretion of catecholamines in a dose dependent manner, this occurred through the α3, α5, and α7 nAChR subunits while α4 nAChR was not involved, and the catecholamines secreted in response to nicotine resulted in increased proliferation [52,53]. These pathways are outlined in Figure 1. All four nAChR subunits, α3, α4, α5, and α7 were found to be upregulated in response to chronic nicotine treatment [53]. Signaling proteins ERK1/2, CREB, Src, and AKT, which are frequently altered in pancreatic cancer, were phosphorylated and thus activated in response to both acute and chronic nicotine treatment, and this could be abrogated by treatment with the β-AR agonist propranolol. Additionally, nicotine could induce proliferation of normal pancreatic cells and pancreatic cancer cells, and this effect could be abrogated by treatment with isopropranolol or the α7 nAChR inhibitor α-bungarotoxin [52,53]. This suggests that nicotine-mediated induction of catecholamine secretion through nAChRs results in activation of β-ARs, which in turn activate a number of tumor promoting signaling pathways, and that nicotine-mediated proliferation could occur through both nAChRs and β-ARs [52,53]. Further evidence for the role of nicotine-mediated induction of an autocrine catecholamine loop resulting in enhanced proliferation of pancreatic tumors was obtained in a mouse xenograft model where it was found that mice treated with nicotine had increased circulating levels of the catecholamines—adrenaline and noradrenaline—as well as systemic cAMP, which coincided with increased xenograft size and increased protein levels of cAMP, p-ERK1/2, and p-CREB [54].

Additional studies have also found nicotine to enhance pancreatic cancer cell proliferation through the activation of ERK1/2 in the MAPK pathway, a pathway known to facilitate proliferation and survival in a broad range of cancer types [45,55,56]. In a study using AR42J stable rat pancreatic cancer cell line, it was found that treatment with nicotine induced proliferation through phosphorylation of ERK1/2 and induced its translocation into the nucleus; however, there was no effect seen on other MAPK target proteins including c-Jun NH2-terminal kinase ½, and p38 MAPK [55,56]. It was further demonstrated that nicotine treatment enhanced acinar cell secretion of amylase, which is of interest as it is known that AR42J cells stimulated to secrete enzymes result in enhanced proliferation; however, this secretion occurred independently of ERK1/2 activation [55,57].

Another mechanism by which nicotine has been shown to promote proliferation is through the inhibition of the inhibitory neurotransmitter GABA [53]. GABA has been shown to inhibit pancreatic tumor growth *in vivo* as well as *in vitro* [54,58], and has more recently been shown to be secreted by PDAC epithelial cells as well as pancreatic ductal cells, contrary to the popular belief that only pancreatic islet cells are capable of GABA secretion [53,59]. Interestingly, treatment with nicotine resulted in a time-dependent decrease in GABA synthesis and secretion in both normal and cancer cells and this was facilitated through the α4 nAChR subunit as well as through the repression of the GABA synthesizing isozymes GAD65 and GAD67 [53]. Thus, nicotine can relieve the GABA-mediated growth inhibition of pancreatic cancer thereby promoting its growth. In the same context, if cells are then treated with GABA, the proliferative effects of nicotine could be abrogated [53]. These findings were confirmed in a mouse xenograft model of pancreatic cancer where mice which were administered nicotine had increased xenograft size, decreased levels of GABA as well as GAD65 and GAD67, the additional treatment with GABA could abrogate the effects of nicotine and inhibit development of xenografts altogether in non-nicotine treated mice [54].

In addition to the proliferative effects of nicotine, it has also been shown that nicotine enhances migration and invasion of pancreatic cancer cells. One mechanism by which this has been shown to occur is through the induction of the mucin MUC4 [19]. Mucins are known to promote tumor initiation and progression through their effect on cell growth and invasion, and are also thought to play an important role in cell-cell and cell-matrix interactions [60,61,62]. Mucins are additionally known to be secreted in association with nicotine uptake in the pancreas [63]. Further, MUC4 in particular is increased in pancreatic adenocarcinomas while remaining undetectable in normal pancreas, and its expression increases progressively in PanINs [64,65]. In a study done on CD18/HPAF pancreatic cancer cell line, it was found that cigarette smoke extract as well as nicotine treatment increased MUC4 expression in a dose dependent manner, and this occurred through the activation of the α7 nAChR and subsequent phosphorylation/activation of JAK2, STAT3, and ERK1/2 [19]. Inhibition of α7 nAChR or ERK1/2, or depletion of STAT3 could abrogate nicotine-mediated induction of MUC4, indicating that nicotine-mediated induction of MUC4 involves cooperative MAPK and JAK/STAT pathway signaling downstream of α7 nAChR [19]. Further, it was observed that nicotine enhanced the migratory ability of CD18/HPAF cells in wound healing assays and this could be abrogated by downregulation of MUC4 [19]. In support of these findings, a mouse xenograft model demonstrated that pancreatic tumor tissues exposed to cigarette smoke had increased levels of α7 nAChR, pSTAT3, and MUC4; and an orthotopic mouse model demonstrated that mice exposed to cigarette smoke at both low and high concentrations had increased pancreatic tumor size and metastasis [19]. In a separate study on multiple pancreatic cancer cell lines, the MUC4 promoter was found to be induced by E2F1 and STAT1 transcription factors, and the association of these factors with the promoter was enhanced in response to nicotine treatment [66]. IFNγ and Retinoic Acid are also known to induce expression of MUC4 in pancreatic cancer and evoke synergistic effects resulting in enhanced induction of target genes [67]. In the context of nicotine, IFNγ and retinoic acid were found to have synergistic or additive effects respectively, on the binding of E2F1 and STAT1 transcription factors to the MUC4 promoter resulting in increased MUC4 expression; further, E2F1 and STAT1 were found to be necessary for this increased expression [66]. Mechanistic studies demonstrated that the nicotine-mediated induction of MUC4 occurred through activation of Src, ERK, and AKT pathways, as well as phosphorylation of Tyk2 and STAT1, but not for other JAK family members [66]. It was additionally found that nicotine increased both proliferation and invasion of pancreatic cells in accordance with other reports in the literature, and MUC4 was found to be necessary for this effect [66].

Nicotine has also been found to promote pancreatic cancer cell proliferation and metastasis through induction of osteopontin (OPN) synthesis and secretion [68]. OPN is a secreted phosphoprotein frequently found to be overexpressed in multiple cancer types, and has been reported in high levels in PDAC [69]. It is known to confer a migratory and tumorigenic phenotype on cancer cells through activation of signaling cascades involved in cell proliferation, invasion, metastasis, and survival [70]. In a study done on three pancreatic cancer cell lines it was found that treatment with nicotine resulted in increased proliferation and concomitant OPN gene promoter activation, mRNA expression, and protein secretion; and this occurred via nAChR receptors, as evidenced by the ability of a general nAChR inhibitor, mecamylamine, to abrogate the nicotine-mediated effect [68]. Mechanistic studies revealed that nicotine-induced expression of OPN occurred through the phosphorylation of ERK1/2, but not other MAPK members p38 or JNK/SAPK, and this activation was required for both nicotine-mediated increase in OPN as well as proliferation [68]. Further supporting these findings, rats exposed to cigarette smoke showed a dose-dependent increase in OPN in the pancreas corresponding to increased nicotine levels in the pancreas and plasma [68]. Additionally, there was a noted increase in OPN levels in PDAC lesions compared to non-malignant or pre-malignant lesions in a cohort of human patients with invasive disease, the majority of whom were smokers; interestingly the presence of OPN was seen not only in the malignant ducts, but also in the normal pancreatic acini surrounding these ducts [68]. In an additional study, OPN was further demonstrated to play a role in nicotine-mediated migration, invasion, and metastasis of pancreatic cancer cells [71]. Here it was reported that nicotine could induce matrix metallaproteinase-9 (MMP9) as well as vascular endothelial growth factor (VEGF), both known to play roles in metastasis; and this was seen at the transcriptional, mRNA, and protein levels [71]. MMP9 in particular is known to play a role in both tumor invasion through matrix degradation as well as angiogenesis, and VEGF plays a crucial role in the induction of new blood vessel formation in angiogenesis promoting tumor growth and survival [72,73]. Interestingly, blocking of OPN using siRNA or antibodies resulted in complete abrogation of nicotine-mediated induction of MMP9 and VEGF promoter activity and mRNA expression suggesting OPN to be a mediator of this effect [71]. It was further demonstrated that OPN itself could induce MMP9 and VEGF in a dose dependent manner, and all three proteins co-localized in PDAC cells as well as in patient tumor samples from smokers. Further, invasive lesions from patients who were smokers had higher levels of MMP9 and VEGF compared to non-smokers [71].

In addition to the pathways discussed above, it has also been shown that nicotine promotes pancreatic cancer cell proliferation and invasion via a Src-ID1 signaling axis [20]. Here it was shown in multiple pancreatic cancer cell lines that nicotine treatment resulted in activation of α7 nAChR and subsequent activation of p-Src kinase, which ultimately lead to the induction of the inhibitor of differentiation-1 (ID1) transcription factor that is known to play a role in cell growth, senescence, and differentiation; and ID1 was required for nicotine-mediated proliferation of pancreatic cell lines [20,74]. ID1 is known to bind to and inhibit transcription factors resulting in an oncogenic phenotype, and has been further correlated with angiogenesis in human patient samples [75]. In an orthotopic mouse model of pancreatic cancer using a metastatic L3.6pl cell line, nicotine could enhance not only tumor growth but also metastasis to the liver; this effect could be abrogated by depletion of ID1 [20]. In patient tumor samples, ID1 expression also was found to correlate with Src activation, poor tumor differentiation, and reduced overall survival [20]. This suggests the Src-ID1 axis to be an important component in nicotine-mediated tumor promotion as well as a clinically relevant prognostic marker.

### 2.3. Calcium Signaling, Smoking and Pancreatic Cancer

Given that nAChRs can activate calcium signaling in various cancer cells, it is highly likely that calcium channels and calcium mediated signaling events promote the growth of various cancers, including that of the pancreas. While aberrant calcium signaling has been associated with pathophysiology of cancer, very little is known about its role in PDACs. Studies in Panc1, MiaPaCa2 and BxPC3 PDAC cells have shown that treatment with the L-type calcium channel blockers nifedipine, diltiazem and verapamil significantly inhibit cell proliferation, possibly through inhibition of calcium influx and modulation of calcium homeostasis [76]. Panc1 cells are known to express functional voltage gated calcium channels, whereas their expression seems to be absent in MiaPaCa2 and BxPC3 cells. But these cells show reduced cell proliferation in response to the calcium channel blocker treatment and it was found that a calcium-activated potassium channel might be playing a role in the case of MiaPaCa2 and BxPC3 cells. Another study analyzing calcium channel inhibitors on cancer cell proliferation showed that the L-type calcium channel blocker fendiline, which belongs to the non-dihydropyridine class, inhibits proliferation of PDAC cells that carry activating mutations in K-Ras (MiaPaCa2, MOH, HPAC and MPanc96) by affecting Ras localization and downstream signaling, while cells that do not carry a Ras mutation (BxPC3) are not affected [77]. Fendiline and other calcium channel blockers (CCBs), such as nifedipine, verapamil, isradipine, diltiazem or the store operated calcium channel blocker SKF96365 can elicit a significant cytotoxic effect on cancer cells. Nifedipine and isradipine belong to the dihydropyridine class, fendiline and verapamil are non-dihydropyridines and diltiazem is a benzothiazipine. Although the potencies of these drugs are comparable, studies have shown their mode of action might be different; fendiline is a slow acting drug with chronic effect while the others are fast acting drugs [78,79]. Irrespective of the mode of action, calcium channel blockers seem to inhibit cancer cell proliferation, implying that calcium dysregulation plays a fundamental role in pancreatic cancer cell proliferation and this aspect deserves further examination.

### 2.4. Calcium Channels and Pumps Associated with PDAC Cell Proliferation and EMT

**Figure 2:**
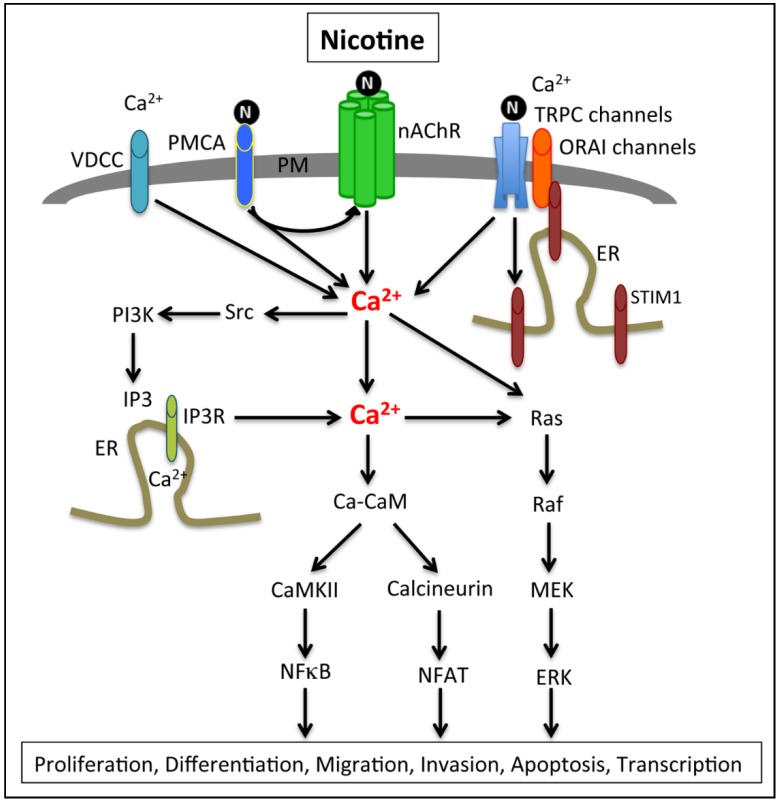
Activation of nAChRs, especially α7-nAChRs, are known to enhance permeability of calcium by directly influencing calcium influx or by activating voltage-dependent calcium channels (VDCC) and by calcium-induced calcium release from endoplasmic reticulum through inositol (1,4,5)-triphosphate receptors (IP3R). Similarly, the plasma membrane calcium ATP-ase (PMCA) activation enhances nAChR-mediated calcium elevations. Furthermore, Src-dependent PI3K activation can enhance IP3 and IP3R-mediated calcium increase and downstream signaling via the Ras-MAPK axis. The increased calcium can also induce calmodulin (CaM)-dependent signaling mechanisms, such as activation of CaM-dependent kinases (CaMKII) or the phosphatase calcineurin. Calcineurin-mediated dephosphorylation of NFAT leads to its nuclear translocation and activation. Nicotine also enhances intracellular calcium levels through activation of the transient receptor potential channels (TRPC), which in turn enhance store operated calcium entry through activation of STIM and ORAI. Studies in PDAC cells have shown that gemcitabine treatment enhances ORAI and STIM activation and their downregulation enhances the gemcitabine-mediated cytotoxicity in cancer cells.

Calcium homeostasis plays an important role in survival and normal functioning of cells and maintenance of calcium homeostasis is achieved by various calcium channels or pumps associated with the plasma membrane [80,81]. It has been suggested that unlike normal cells, which use mitochondrial metabolism as the major energy source, cancer cells mainly use glycolytic ATP as the energy source [82,83,84,85,86,87,88]. The glycolytic ATP enhances calcium pumps known as plasma membrane calcium ATPase (PMCA) to provide the energy necessary for maintenance of low calcium levels in pancreatic cancer cells [89]. This shift in energy source enhances survival of cancer cells even under hypoxic conditions. Excess intracellular calcium lead to cytotoxicity in cancer cells and it has been shown that the ubiquitously expressed PMCA plays an important role in maintenance of the normal levels of intracellular Ca [90,91]. Studies performed in Panc1 and MiaPaCa2 cells treated with a glycolytic inhibitor 3-bromopyruvate (BrPy) or a protonophore (CCCP) that collapses mitochondria showed that inhibition of glycolysis, but not mitochondrial metabolism, induces severe ATP depletion and PMCA inactivity, thereby increasing the cytosolic calcium load, and enhanced cell death. This study therefore suggests that PMCA plays a crucial role in resistance and survival of PDAC cells by promoting calcium homeostasis at the expense of glycolytic ATP, and targeted inhibition of PMCA function would be beneficial for treatment of PDAC. The pathways involved are depicted in Figure 2. Studies, unrelated to PDAC, have shown that cigarette smoking affects the PMCA activity [92], therefore it is possible that nicotine-mediated changes in pancreatic acinar cell PMCA contributes to pancreatic diseases as well. Functional proteomic studies on rat brains have shown that α7 nAChRs are associated with plasma membrane calcium-ATPase pump isoform 2 (PMCA2) [93]. Analysis of cultured hippocampal interneurons showed a role for PMCA2 in nAChR-mediated calcium elevations in these cells and PMCA2 inhibition was associated with loss of α7-nAChR clusters [93]. This suggests that activation of PMCA induces nAChR clustering and signaling, which might have consequences on transformed cells. It is possible that in PDAC, PMCA-mediated regulation of nAChR plays a role in proliferation, migration or invasion of cancer cells.

Non-excitable cells are known to express store operated calcium entry (SOCE) associated channels, which seem to play a major role in calcium entry in these cells [94]. These channels are activated upon depletion of intracellular calcium stores. Recent studies have shown that the stromal interacting molecule 1 (STIM1) and ORAI1 play an important role in SOCE-associated calcium influx in cells [95]. ORAI1 is the pore-forming unit of SOCE, and STIM1 interaction with the cytoplasmic domains of ORAI channels have been shown to activate them [96]. Several studies have shown that ORAI1 and STIM1 play a role in proliferation, migration and invasion of cancer cells [95,97,98]. In addition to the SOCE channels, the transient receptor potential channels (TRP) also seem to play an important role in various types of human cancers, including pancreatic cancers [99,100,101,102,103,104,105,106,107]. TRP channels are plasma membrane associated cation channels, which are permeable to calcium, sodium and magnesium. The TRP superfamily consists of 7 subfamilies, namely TRPC (TRP-Canonical), TRPV (TRP-Vanilloid), TRPM (TRP-Melastatin), TRPN (TRP-NompC), TRPA (TRP-Ankyrin), TRPP (TRP-Polycystin) and TRPML (TRP-MucoLipin) [107]. Among these, the TRP-melastatin (TRPM) subfamily has been associated with progression of PDACs. It has been shown that the TRPM7 and TRPM8 channels are required for the normal and oncogenic development of exocrine pancreas [108].

Recent studies by Yee and colleague [109,110] showed that TRPM7 and TRPM8 channels are aberrantly overexpressed in PDAC tissues and play a role in proliferation of PDAC cells. To determine the role of TRPM8 in PDAC cells these authors downregulated its expression in Panc1 and BxPC3 cells and found that the cells undergo replicative senescence, as indicated by presence of flat cells with multiple micronuclei and multilobed, partially fragmented nuclei. MTS assay and cell counting indicated that TRPM8 knockdown led to a reduction in proliferative potential of the PDAC cells. Additionally, FACS analysis of the TRPM8 knockdown cells showed accumulation of cells in G0/G1 with a corresponding decrease in S and G2 phase, indicative of reduced proliferation compared to the control siRNA transfected cells. The cells downregulated for TRPM8 showed an increase in expression of p21 and p27 mRNA, with no changes in G1 cyclins or cyclin B1, indicative of cell cycle arrest. These data therefore imply that calcium dysregulation and TRPM8 activation plays a role in PDAC cell proliferation contributing to tumor growth and an inhibition of calcium activation could therefore interfere with the TRPM8-mediated cancer cell proliferation and tumor promotion. Studies from the same group have shown that, similar to TRPM8, siRNA-mediated downregulation of TRPM7 also induces replicative senescence and in this case the expression of p16 and the Werner’s syndrome gene are induced [111].

A recent study comparing the PDAC cells and the normal human pancreatic ductal epithelial (HPDE) cells showed that anoctamin 1 (ANO1), also known as transmembrane member 16A (TMEM16A), a voltage sensitive calcium-activated chloride channel expression is specifically increased in cancer cells. These studies were carried out using Panc-1, Mia PaCa 2, Capan-1, AsPC-1 and BxPC-3 PDAC cells, which showed a significant increase in both mRNA and protein levels in ANO1. ANO1 inhibitors or RNAi-directed knockdown showed significant decrease in migration but not proliferation of cancer cells implying that calcium aberrancies contribute to EMT in cancer cells [112].

A connection between nicotine, store operated calcium entry (SOCE) and TRPC channels have been drawn from studies in rats and *C. elegans*. An analysis of rats exposed to cigarette smoke had shown that the intracellular calcium levels and SOCE (store operated calcium entry) levels were significantly increased in the distal pulmonary arterial smooth muscle cells (PASMCs) compared to that in rats that were not exposed to cigarettes [113]. This was associated with an increase in the expression of the TRPC (transient receptor potential canonical) channels TRPC1 and TRPC6. Similar results were obtained with cultured PASMCs treated with 10 nM nicotine and RNAi-directed knockdown of the TRPC channels inhibited the increase in intracellular calcium and SOCE levels implying that these increases were TRPC-dependent. Similarly, studies in *C. elegans* have shown that nicotine affects their behavior, which is interrupted by mutations in TRPC channels. Overexpression of a human TRPC channel rescued the defects revealing the important role of the TRPC channels, especially TRP-1 and TRP-2, on nicotine-dependent behavioral changes [114,115]. These studies therefore suggest that TRPC might play a role in nicotine-mediated changes in humans, contributing to nicotine addiction [116] or calcium-dependent signaling events (intracellular calcium levels and SOCE levels), thus contributing to pathologic conditions, including development of PDAC. Understanding the underlying calcium-dependent mechanisms and their correlation with exposure to tobacco smoke might unravel novel signaling pathways and crosstalks that can potentially be targeted for therapy.

### 2.5. nAChRs and Immune Modulation of PDACs

**Figure 3:**
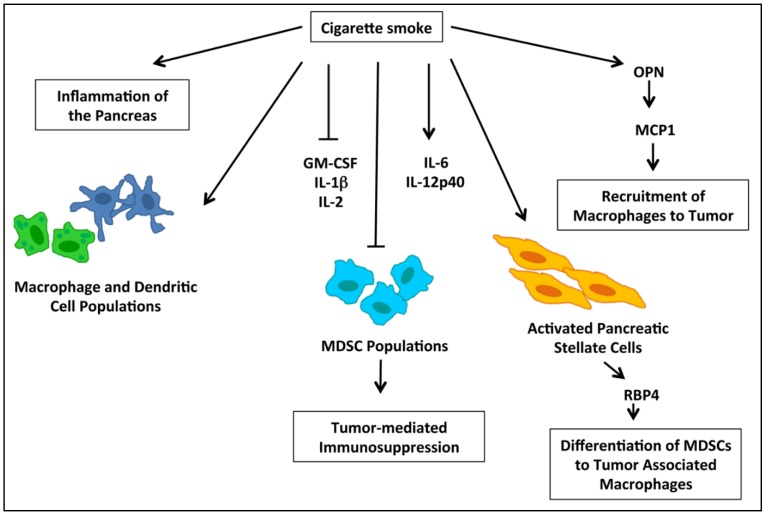
A schematic representation of the modulation of immune response by cigarette smoke leading to tumor promotion. Cigarette smoke leads to general inflammation of the pancreas as well as increased production of immune cytokines such as IL-6 and IL-12p40, while suppressing cytokines such as IL-1β, IL-2, and GM-CSF. Further, cigarette smoke enhances macrophage and dendritic cell populations, but results in a decrease in Myeloid Derived Suppressor Cell (MDSC) populations. Cigarette smoke also stimulates recruitment of macrophages to pancreatic cancer tumors through activation of osteopontin (OPN) which in turn activated Macrophage-Chemoattractant Protein-1 (MCP1).

The tumor microenvironment plays a critical role in both the development and progression of cancer, and accumulating evidence has demonstrated the importance of immune response in this context. Myeloid derived suppressor cells (MDSCs) are thought to play a role in tumor-mediated immunosuppression, and are detected in the circulation during pre-neoplastic stages of pancreatic tumor development [117,118]. These cells assist tumors in evading immune surveillance by suppressing T-cells while promoting T-regulatory cell development [118]. Nicotine is known to modulate immune response in other cancer types, specifically lung cancer; and in a recent study using a KRAS G12D model of pancreatic cancer, cigarette smoke was found to reduce systemic accumulation of granulocytic and monocytic MDSCs while increasing macrophage and dendritic cell populations [46]. It was further shown that mice exposed to cigarette smoke had higher levels of the cytokines IL-6 and IL-12p40, while there were reduced levels of GM-CSF, IL-1beta, and IL-2, and no differences were reported for G-CSF, M-CSF, IL-10, IL-13, TGFβ, TNFα, or MIP1α [46]. The molecular events involved in the modulation of the immune response by cigarette smoke are depicted in Figure 3. Cigarette smoke further resulted in increased levels of inflammatory markers in the pancreas associate with overall inflammation of the pancreas, and this was accompanied by an increase in activated pancreatic stellate cells, which secrete serum retinoic acid binding protein 4 which contributes to the differentiation of MDSCs to tumor-associated macrophages (TAMs) [46]. TAMs additionally enhance expression of HB-EGF in pre-neoplastic pancreatic lesions, and this protein is associated with/facilitates acinar-to-ductal metaplasia [46]. In an additional study investigating the impact on inflammation and immune response, nicotine was shown to enhance expression of monocyte-chemoattractant protein 1 (MCP1), which is produced by a number of cell types in the tumor microenvironment and acts to recruit macrophages to tumor sites [119]. Nicotine induction of MCP1 was found to occur through OPN, which is also known to be induced by nicotine [119]. In invasive pancreatic cancer patient samples, MCP1 was found to be expressed in 60% of the cases and the majority of samples assessed were from smokers; further, MCP1 colocalized with OPN in invasive patient tissue samples [119]. Overall, these studies demonstrate that smoking can modulate the immune response to promote tumor growth.

### 2.6. Role of nAChR and Calcium Signaling in Therapeutic Resistance of PDACs

The standard of care for advanced PDAC has been gemcitabine or gemcitabine in combination with other agents, but this approach has not proven effective in increasing survival, primarily due to its highly drug resistant nature [1,3]. Nicotine has been shown to facilitate chemoresistance in multiple cancer types including pancreatic cancer, [20,30,120] and signaling molecules involved in the progression of pancreatic cancer have been shown to play a role in drug resistance [121]. Nicotine was found to increase the viability of L3.6pl cells, which were sensitive to gemcitabine treatment, as well as L3.6pl cells that had acquired resistance to gemcitabine [20]. These results were confirmed in an orthotopic xenograft experiment using L3.6pl cells; mice which were administered nicotine had increased tumor size and were resistant to treatment with gemcitabine, whereas their non-nicotine treated counterparts had smaller tumor size and responded well to treatment [20]. The ability of nicotine to essentially reverse the sensitivity of gemcitabine sensitive cells suggest that nicotine has a cytoprotective effect and could potentially reduce sensitivity to chemotherapy in PDAC patients [20]. Further, it was demonstrated that depletion of the ID1 transcription factor, which is induced by nicotine downstream of Src, resulted in increased sensitivity to gemcitabine in pancreatic cancer cell lines with both innate and acquired resistance, suggesting that ID1 facilitates nicotine-mediated chemoresistance [20]. In an additional study investigating the effect of chronic exposure to moderate levels of nicotine on therapeutic outcome of pancreatic cancer it was found that treatment of BXPC-3, a gemcitabine sensitive cell line, with nicotine for 7 days increased the concentration of gemcitabine necessary to reduce cell viability [122]. This reduction in gemcitabine sensitivity was shown to be mediated through increased levels of pSrc, pAKT, and pERK; levels of cleaved caspase 3 were significantly reduced in nicotine pre-treated cells, indicating reduced cell death in response to gemcitabine treatment [120,123]. Further, in a mouse xenograft model using BXPC-3 cells, mice which were chronically exposed to moderate levels of nicotine had reduced response to gemcitabine treatment compared to non-nicotine treated mice, and analysis of tumor tissues showed that the induction of cleaved caspase 3 by gemcitabine was completely abrogated upon nicotine treatment coinciding with increased levels of pSrc, pAKT, and pERK [123]. These results indicate that chronic exposure to moderate levels of nicotine, could significantly reduce the efficacy of gemcitabine *in vitro* and *in vivo*. This raises the possibility that patients who may be achieving smoking cessation through prolonged nicotine replacement might be less responsive to chemotherapeutic regimens. Interestingly, further studies have demonstrated that treatment with GABA, which has been shown to reduce pancreatic tumorigenecity and reverse nicotine-mediated tumor promotion, could additionally reverse nicotine-induced resistance to gemcitabine in mouse xenograft models [123]. Treatment with GABA could reverse the nicotine-mediated increase in cAMP formation as well as pCREB, pSrc, pAKT, and pERK levels, and could reverse the nicotine-mediated decrease in capase 3 cleavage after treatment with gemcitabine [120]. Furthermore, GABA reversed the nicotine-mediated induction of proteins involved in tumor growth and metastasis including MMP9, MMP2, and EGR1. This suggests that GABA may be an effective therapeutic agent for pancreatic cancer to overcome gemcitabine resistance conferred by nicotine in patients with a history of smoking.

In addition to enhanced cancer cell proliferation and promotion of EMT, it appears that aberrant calcium signaling also induces drug resistance in pancreatic cancer cells. A recent study showed that PDAC cells express STIM1 and ORAI1 and their expression is associated with drug resistance in these cells [124]. Here they used Panc1, AsPC1, BxPC3, Capan1, MiaPaCa2 and the immortalized H6C7 normal pancreatic ductal epithelial cells in their analysis and showed that all of these cells express ORAI1 and STIM1 mRNA and protein at varying levels, with Panc1 showing the highest levels of both ORAI1 and STIM1 expression. In this study, RNAi-directed downregulation of STIM1 and ORAI1 in Panc1 cells led to a significant reduction in intracellular calcium levels. Here they depleted intracellular calcium using thapsigargin in cells cultured in calcium-free solution and induced SOCE-mediated calcium influx by addition of extracellular calcium. Then they examined the effect of 5-fluorouracil or gemcitabine on Panc1 cells transfected with siRNA to ORAI or STIM1 or a combination of both siRNAs and found that the SOCE regulator transfected cells show a significant increase in cytotoxicity compared to control siRNA transfected cells. Additionally, their studies using 5-FU or gemcitabine on control siRNA transfected cells showed that expression of ORAI1 and STIM1 was significantly increased and this was associated with enhanced SOCE-mediated calcium entry into these cells. These studies imply that the drug resistance observed in PDAC could be brought about by aberrancies in calcium signaling mediated through enhanced expression of the SOCE associated STIM1 and ORAI1 and targeted inhibition of these regulators may enhance sensitivity of PDACs to the established treatment modalities.

## 3. Conclusions

Tobacco smoke has been highly correlated with pancreatic cancer initiation and progression, in a dose and concentration dependent manner. While nicotine, the addictive component of tobacco smoke, cannot initiate tumorigenesis of the pancreas, the studies discussed here demonstrate that it can promote proliferation, migration, and invasion of cancer cells as well as growth and metastasis in mouse models. Further, it appears that aberrant calcium signaling plays an important role in proliferation, migration, invasion, angiogenesis or metastasis of PDAC. This involves various calcium channels, calcium pumps or calcium binding proteins, which are known to be affected by exposure to cigarette smoke or nicotine. Therefore, it is possible that nicotine or cigarette smoke-induced cancer cell proliferation, migration, invasion or tumor growth and metastasis associated with PDAC is at least partly brought about by aberrant calcium signaling. Targeting pathways involved in these nAChR-mediated as well as calcium-mediated events might be beneficial in overcoming smoking-related growth and progression of PDACs and would have high clinical utility to improve therapeutic outcome.
